# The role of PDGF in radiation oncology

**DOI:** 10.1186/1748-717X-2-5

**Published:** 2007-01-11

**Authors:** Minglun Li, Verena Jendrossek, Claus Belka

**Affiliations:** 1 Department of Radiation Oncology, University Hospital Tuebingen, Germany

## Abstract

Platelet-derived growth factor (PDGF) was originally identified as a constituent of blood serum and subsequently purified from human platelets. PDGF ligand is a dimeric molecule consisting of two disulfide-bonded chains from A-, B-, C- and D-polypeptide chains, which combine to homo- and heterodimers. The PDGF isoforms exert their cellular effects by binding to and activating two structurally related protein tyrosine kinase receptors. PDGF is a potent mitogen and chemoattractant for mesenchymal cells and also a chemoattractant for neutrophils and monocytes. In radiation oncology, PDGF are important for several pathologic processes, including oncogenesis, angiogenesis and fibrogenesis. Autocrine activation of PDGF was observed and interpreted as an important mechanism involved in brain and other tumors. PDGF has been shown to be fundamental for the stability of normal blood vessel formation, and may be essential for the angiogenesis in tumor tissue. PDGF also plays an important role in the proliferative disease, such as atherosclerosis and radiation-induced fibrosis, regarding its proliferative stimulation of fibroblast cells. Moreover, PDGF was also shown to stimulate production of extracellular matrix proteins, which are mainly responsible for the irreversibility of these diseases. This review introduces the structural and functional properties of PDGF and PDGF receptors and discusses the role and mechanism of PDGF signaling in normal and tumor tissues under different conditions in radiation oncology.

## Background

PDGF was originally identified as a constituent of whole blood serum that was absent in cell-free plasma [1,2] and subsequently purified from human platelets [3,4]. Although the α-granules of platelets are a major storage site for PDGF, recent studies have shown that PDGF can be synthesized by a number of different cell types such as macrophages, epithelial and endothelial cells [5-8]. Studies have shown that PDGF has important physiologic functions in organ development [9,10]. PDGF has also been implicated in a wide variety of pathological processes, including fibrosis, atherosclerosis, glomerulonephritis and aggressive fibromatosis [11-15]. Moreover, aberrant production of PDGF and autocrine stimulation may be an important mechanism in the neoplastic conversion of PDGF receptor-positive cells [16-18]. Here, we point out the most important features of PDGF and PDGF receptors concerning their roles in radiation oncology.

## PDGF structure and signaling

PDGF is a disulfide-linked dimer of two related polypeptide chains, designated A, B, C and D, which are assembled as heterodimers (PDGF-AB) or homodimers (PDGF-AA, PDGF-BB, PDGF-CC and PDGF-DD) [19-21]. PDGF exerts its biological activity by binding to structurally similar PDGF receptors (PDGFR-α and -β). The PDGFR-α binds to A-, B- and C- chains with high affinity, whereas PDGFR-β only binds the B- and D- chains [22-25]. Different from PDGF-A and -B, PDGF-C and PDGF-D require proteolytic activation before binding to and activation of PDGFR [19,20]. PDGF ligand dimer induces dimerization of both receptors and subsequently autophosphorylation of the PDGF receptor tyrosine kinase (RTK). Activated RTK phosphorylates numerous signaling molecules that initiate intracellular signaling cascades (Reviewed in Ref. [31]).

The best characterized mechanisms by which PDGF down-streaming signaling mediates cellular responses involve the activation of the ras/MAPK pathway, which can functionally increase cellular proliferation, migration and differentiation [26], and the PI3K/Akt pathway, which promotes cell survival [27]. Both pathways are of crucial importance for tumor resistance to radiotherapy and chemotherapy. Furthermore, platelet-derived growth factor (PDGF) exerts its potent mitogen and chemotactic effects in a variety of mesenchymal cells such as fibroblasts, vascular smooth muscle cells, glomerular mesangial cells, and brain glial cells [14,28-30] making PDGF a potential key molecule for tissue rebuilding in response to physiological and non-physiological conditions.

## PDGF in oncology

Many investigators have shown that autocrine activation of PDGF was interpreted to be an important pathogenetic mechanism involved in different brain tumors [16-18].

In gliomas, analysis of PDGF/PDGFR expression suggested the presence of autocrine and paracrine loops of PDGF in glioma activating PDGFR-α in glioma cells, while PDGFR-α expression was higher in malign gliomas than in benign gliosis [17].

Moreover, the recently identified new PDGF isoforms, PDGF-C and -D are also detectable in glioblastoma cell lines and primary human tumor tissues [31].

On the other hand, treatment with a PDGFR antagonist interrupted autocrine growth stimulation and thus inhibited survival and mitogenesis in glioblastoma cells and prevented glioma formation in a mouse xenograft model [31,32].

In the case of meningioma, Adam and his colleagues provided evidence that cytokines secreted by meningioma cells profoundly stimulated growth of meningioma and neuroblastoma cells in vitro, while this growth stimulation was completely abolished by a neutralizing antibody against PDGF [16]. Todo et al showed DNA synthesis in tumor cells could be inhibited through an antagonist of PDGF in three of seven meningiomas cell lines [32].

Similarly, autocrine loops involving PDGF-A or -B and their respective receptors was also observed in many malignant and low-grade astrocytomas, while the activation of PDGF autocrine loops was suggested to be an early event in the pathogenesis of malignant astrocytomas [33].

Aggressive fibromatosis also referred to as desmoid tumor develops from muscle connective tissue, fasciae and aponeuroses. The neoplasm is composed of fibrocyte-like cells, and characterized by local infiltrative growth and high risk of recurrence (~70%) after surgical treatment [34]. Depending on the location and extent of the tumor, radiotherapy is indicated for patients with unresectable tumors or those with positive resection margins. Overexpression of PDGF were observed in desmoid tumors, while inhibition of PDGF signaling by imatinib induced overall 1 year tumor control rate of 36.8% in a phase II clinical study [15]. Thus, inhibition of PDGF may be an attractive therapy option, alone or combined with surgery or/and radiotherapy in refractory cases.

Another example for an important role of PDGF in oncogenesis is the so-called gastrointestinal stromal tumors (GISTs). Many GISTs have gain-of-function mutations of c-kit receptor tyrosine kinase (KIT) gene. Approximately 35% of GISTs lacking KIT mutations have intragenic activation mutations in PDGFR-α [35].

However, the alternative defects lead to similar alterations of the downstream signaling cascades and cytogenetic changes. Therefore the defects (gain-of-function through mutated-KIT or mutated-PDGFR-α) appear to be alternative and mutually exclusive [35].

Likewise, overexpression of PDGF and c-kit was also observed in Leydig tumors. Treatment with imatinib almost completely inhibited Leydig tumor growth in an allograft mouse model by inhibition of PDGF and c-kit signaling with no drug-resistance development during imatinib treatment [36].

The clinical success of imatinib/gleevec, a triple tyrosine kinase inhibitor of c-kit, PDGF and c-Abl signaling, in chronic myeloid leukemia [37] and gastrointestinal stromal tumors [38] has accelerated the development of molecular targeted cancer therapy. It is highly likely that many more antitumoral substances of this class will be developed and discovered in the near future.

## PDGF and angiogenesis

In addition to its direct tumor growth promoting effect, the importance of PDGF in tumor propagation relates to the inherent angiogenic activity [39]. In this regard, PDGF has been shown to be essential for the stability of normal blood vessel formation by recruiting pericytes and smooth muscle cells [40]. PDGF-B expression by endothelial cells recruits pericytes through a short-range paracrine mode [41]. Pericytes expressing PDGFRs migrate along steep gradient of PDGF-B in the peri-endothelial compartment to endothelial cells and thus initiate intimate association with the abluminal surface of the endothelial cells [41]. Pericyte-deficiency promotes a range of microvascular changes, such as endothelial hyperplasia, vessel dilation, leakage and rupture, leading to capillary microaneurysms, and lethal microhemorrhage [40]. Despite structural and functional abnormalities in the microvasculature, mice embryos deficient of up to 90% pericytes are compatible with embryonic and postnatal survival, while loss of more than 95% of the pericytes is lethal [40,42]. This suggests that a rather low threshold density of pericytes is required for basal microvascular function.

Angiogenesis is an important event in tumor growth, since tumors located more than 100–200 μm distant from a blood vessel need neovascular formation to ensure a sufficient supply of nutrients and oxygen [43]. Tumor cells in hypoxia secrete cytokines, including VEGF, PDGF, basic fibroblast growth factor (bFGF), insulin growth factor (IGF), to stimulate neovascular formation [43].

However, neovasculature in tumors differs strikingly from normal physiologic vessels. The badly coordinated growth leads to vessel malformation including vessel dilation, tortuosity, leakage, rupture and formation of microaneurysms [40]. Interestingly, these hallmarks of microvascular malformation in tumors were found to be identical with the alterations found in pericyte-deficient mice (PDGF-B -/- or PDGFR-β -/-), pointing to a pericyte-deficiency in the disordered neovascular formation in tumors [41].

Since small numbers of pericytes in tumor vessels may be critical for vessel integrity and function [40], targeting pericytes in tumors may be an attractive and efficacious way for anti-angiogenic therapy.

Recent data from experiments *in vivo *imply that targeting pericytes actually provides additional benefits [44]. Traditionally, endothelial cells as a host component in the tumors with normal genome are suggested to be the primary target for anti-angiogenic therapies [45]. Inhibiting VEGF in endothelial cells reduced endothelial cell survival, proliferation, tube formation and invasion *in vitro *[45]. However, Erber and his colleagues demonstrated that endothelial cells were resistant to the inhibitory effect of SU5416 by blocking VEGFR *in vivo *through pericyte mediated escape strategies via the Ang-1/Tie2 pathway [46]. Combined inhibition of VEGF and PDGF signaling enforces tumor vessel regression by direct anti-angiogenic effect to endothelial cells and pericytes and by inhibiting pericyte mediated endothelial cell survival mechanisms [46].

This view is also supported by other studies showing that tumor vessels lacking pericytes are more dependent on VEGF for their survival than are vessels invested by pericytes [44]. In fact, sorafenib and sunitinib/SU11248 act as anti-angiogenic agents by inhibiting VEGFR-2/-3, PDGFR-β, Flt-3, and c-KIT. Both drugs exert clear clinical effects in patients with renal cell carcinoma which are most likely mediated via anti-angiogenic effects [47,48]. The therapeutic efficacy to other tumors is currently under investigation [48].

In conclusion, PDGF has at least two distinct functions in pro-angiogenic signaling. On the one hand PDGF increases survival and proliferation of endothelial cells and on the other hand, PDGF regulates vessel growth via pericyte recruitment and association to newly formed vessels.

## PDGF inhibition in combination with radiotherapy

Ionising radiation causes miscellaneous effects to the tumor mass. It exerts a direct antitumoral effect on tumor cells, for example through DNA double-strand-break leading to failure of DNA transcription and duplication and eventual death of tumor cells [49]. However, radiation induced damage of endothelial cells plays a major role in tissue damage and antitumoral efficacy [45]. In this regard, within hours after ionising radiation, lesions with structural changes could be observed in endothelial cells by using electron microscopy [50]. Thus, ionising radiation can be also considered as a potent anti-angiogenic agent [45].

On the other hand, it was shown that tumor cells are able to produce pro-angiogenic cytokines including VEGF, PDGF and FGF in response to ionising radiation. These pro-angiogenic cytokines could protect endothelial cells and vessels from radiation-induced damage and consequently ensure supply of oxygen and nutrients for tumor cells [9,11,18]. The secretion of PDGF could also be stimulated in irradiated stromal cells, such as endothelial and fibroblast cells [51]. Elevated expression of these growth factors correlates with higher vessel density and negative clinically prognosis in various tumors [52]. Usually, such tumors possess a relative resistance to radiation therapy [53].

Inhibition of pro-angiogenic signaling by tyrosine kinase inhibitors can therefore augment the radiation induced damage to endothelial cells and abolishes the tumor cells mediated protection. Moreover, these inhibitors can prevent the re-growth of endothelial cells and neovascular formation.

Consequently, anti-angiogenic substances targeting VEGF and PDGF may increase anti-angiogenic activity of ionising radiation and possess a potent antitumoral synergy with radiation.

Glioma is a good example for demonstration of the dual role of PDGF signaling in the oncogenesis and angiogenesis in tumor mass.

Using in situ hybridization and immunohistochemistry techniques, Hermanson et al demonstrated the presence of autocrine and paracrine loops in gliomas, activating the PDGFR-α in glioma cells. The activation of PDGFR-β in endothelial cells was also observed in the tumor mass, pointing to the dual role of PDGF signaling in oncogenesis and angiogenesis in glioma tumors [17].

On the one hand, treatment with imatinib/gleevec disrupted an autocrine PDGF/PDGFR loop by specifically inhibiting phosphorylation of PDGFR and thus exerted a synergistic antitumoral effect with ionising radiation as radiosensitizer [54]. And on the other hand, targeting PDGF signaling inhibits the hypoxia-induced angiogenesis and strengthens the anti-angiogenic effect of radiation [46].

## PDGF in radiotherapy-induced fibrogenesis

The development of acute inflammation and chronic fibrosis is a frequent side effect of ionising radiation and thus a dose-limiting factor for treatment efficacy [55].

In the case of lung tumors, the dose limitation imposed by normal tissue tolerance presently precludes successful radiotherapeutic treatment in many patients [56]. Pulmonary fibrosis is a progressive condition, characterized by mesenchymal cell proliferation, the subsequent deposition of extracellular matrix proteins and extensive remodeling of the pulmonary parenchyma [57]. In both human and animal model systems, acute pneumonitis and late fibrosis are directly dependent upon total irradiation dose, fraction size, and lung volume irradiated [58-60]. New precise radiotherapy techniques can spare more normal tissue around tumor volume and thus reduce the intensity of side effects. However a recent study has shown that 14.6 % patients with lung cancer still developed intermediate grade radiogenic pneumonitis after primary radiotherapy with dose escalation using 3D conformal techniques and 13.8 % patients developed fibrosis [61].

The treatment of fibrosis remains still elusive, since the exact mediators and mechanisms involved in fibrogenesis are not completely understood [57]. The traditional interpretation of radiation-induced fibrosis as a consequence of acute inflammation has been questioned in recent years, because clinical measures of inflammation do not correlate well with fibrotic progression and because anti-inflammatory drugs do not significantly affect clinical outcome [56,62,63]. New evidence suggests that immediate intercellular communications through regulation of cytokines happens within hours to days after irradiation [64].

A number of investigations provided clear evidence for increased expression of various cytokines including PDGF, transforming growth factor-β, tumor necrosis factor-α and interleukin-1 in response to ionising radiation [22,65-67]. In this regard, some pro-inflammatory cytokines seem to be important for the acute impairment in the pneumonitis phase, for example TNF-α and CD95-ligand [66,68], whereas others are involved in the regulation of the fibrotic response. For the development of fibrosis, transforming growth factor-β is till now a widely accepted key player [69].

Moreover, recent evidence supports an important role of PDGF for the development of lung fibrosis in response to ionising radiation. Firstly, PDGF and PDGFR are expressed at low levels in normal adults, while elevated levels are detected in lungs of patients with radiation-induced pulmonary fibrosis [70]. Augmented expression of PDGF is further observed in asbestos-, bleomycin- and idiopathic pulmonary fibrosis [71-73]. Increased expression of PDGF in rat lungs by adenoviral delivery or lung-specific over-expression in mice is associated with pronounced lung fibrosis [74,75]. Moreover, inhibiting the PDGF pathway with neutralising antibodies to PDGF or administration of soluble extracellular region of PDGFR-β could attenuate fibrotic development [76,77].

Recently it has been shown that three distinct receptor tyrosine kinase inhibitors (RTKI), overlapping in inhibition of PDGF signaling, attenuated radiation-induced pulmonary fibrogenesis *in vivo *[78]. The radiation-induced overexpression of PDGF led to phosphorylation and activation of PDGFR in lungs of irradiated mice, while the phosphorylation of PDGFR was strongly inhibited in both irradiated groups treated with RTKIs. Accordingly, the treatment with RTKIs attenuated the development of pulmonary fibrosis in excellent correlation with clinical, histological, and computed tomography results, although the acute inflammatory response induced by radiation injury was not completely abrogated. Moreover, all three tyrosine kinase inhibitors reduced lung fibrosis after radiation injury and prolonged animal survival. Thus, there is hard evidence to support the important role of the PDGF/PDGFR system for mesenchymal cells in proliferative diseases.

Since fibroblasts are the putative effector cells, recruitment and stimulation of fibroblasts should be the most important event during development of fibrosis. In this regard, PDGF may exert profibrotic effect through its mitogenic and chemotactic stimulation to mesenchymal cells, such as fibroblasts, myofibroblasts and smooth muscle cells [79]. Moreover, PDGF was also shown to stimulate production of extracellular matrix proteins, such as collagen, hyaluronic acid, fibronectin and proteoglycan [80-83], which are mainly responsible for the irreversibility of fibrotic lesion.

The radiation-induced secretion of PDGF has been assumed to derive solely from leucocytes. However, radiation of stromal cells, such as fibroblasts and endothelial cells, induced paracrine PDGF in co-culture systems which substantially stimulated the proliferation of non-irradiated fibroblasts [51].

In accordance with these results, endothelial cells were reported as potential sources of PDGF after radiation *in vitro *[84]. Moreover, the expression of c-sis mRNA in epithelial cells was also observed in certain pulmonary fibrotic diseases [85].

Other experiments demonstrated that anti-inflammatory treatment with dexamethasone did not decrease the level of PDGF-BB or the mitogenic activity of bronchial alveolar lavage fluid for fibroblasts in the chronic lung disease of prematurity [86]. Savikko and his colleagues also showed that limiting the extent of inflammation by cyclosporin A treatment did not inhibit the expression of PDGF ligands and receptors [87]. Thus, stromal cells, such as endothelial, fibroblasts cells, should be at least partially responsible for the release of cytokines, including PDGF.

**Figure 1:**
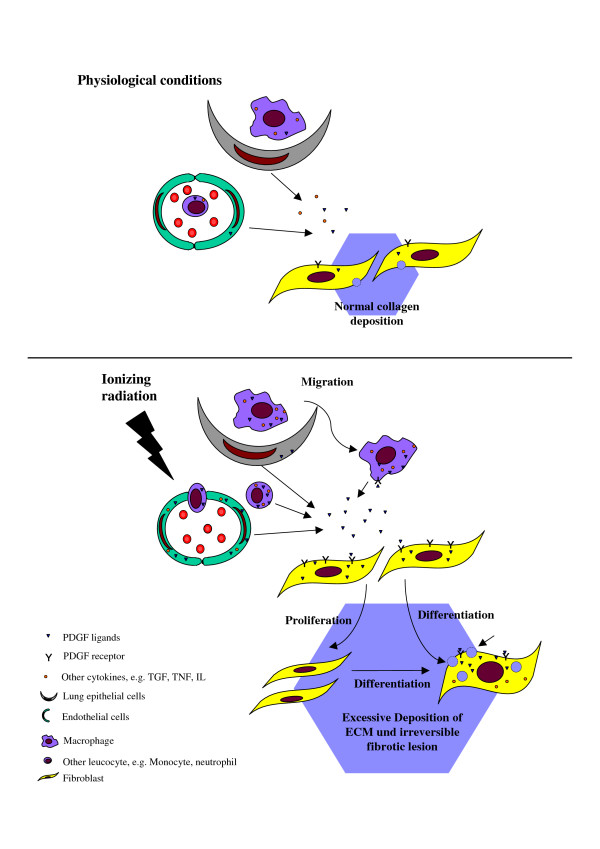
Schematic presentation of radiation induced fibrogenesis in lungs. Illustration of a microenvironment of gas-blood exchange unit in lungs in the physiologic conditions (upper part) and radiation induced activation of PDGF pathways in the fibrogenesis in lungs (lower part).

A schematic diagram depicts the suggested role of radiation induced PDGF signaling in fibrogenesis (Fig. 1).

## Conclusion and outlook

PDGF signaling plays an important role in radiation oncology with respect to its oncogenic, angiogenic and profibrotic effects. The rational of targeting PDGF signaling in radiation oncology can arise in three ways: 1) the direct antitumoral potential, 2) the anti-angiogenic impact, and 3) the antifibrotic activity which protects normal tissue from the side effects of ionising radiation.

Suppression of PDGF is discussed as one potential mechanism of action of some novel antifibrotic drugs undergoing clinical trials [88,89]. It has been suggested that pirfenidone and interferon gamma, both ameliorate lung fibrosis by downregulation of PDGF expression [72,90].

However, since diverse signaling pathways activated by growth factor receptors induce broadly overlapping, rather than independent sets of signaling, it's unlikely to completely inhibit a biologic process by blocking a single cytokine/growth factor. Thus multi-targeted agents may be more effective in the oncological therapy.

At the same time, a special attention should be paid to the side effects of this new class of molecular targeted agents, since clinical experience is still sparse, especially in combination with radiotherapy and chemotherapy.

## Competing interests

The author(s) declare that they have no competing interests.

## Authors' contributions

ML drafted the manuscript. CB and VJ critiqued the manuscript. All authors read and approved the final manuscript.

## References

[B1] Ross R, Glomset J, Kariya B, Harker L (1974). A platelet-dependent serum factor that stimulates the proliferation of arterial smooth muscle cells in vitro. Proc Natl Acad Sci U S A.

[B2] Westermark B, Wasteson A (1976). A platelet factor stimulating human normal glial cells. Exp Cell Res.

[B3] Heldin CH, Westermark B, Wasteson A (1979). Platelet-derived growth factor: purification and partial characterization. Proc Natl Acad Sci U S A.

[B4] Antoniades HN, Scher CD, Stiles CD (1979). Purification of human platelet-derived growth factor. Proc Natl Acad Sci U S A.

[B5] Demayo F, Minoo P, Plopper CG, Schuger L, Shannon J, Torday JS (2002). Mesenchymal-epithelial interactions in lung development and repair: are modeling and remodeling the same process?. Am J Physiol Lung Cell Mol Physiol.

[B6] Zhang S, Smartt H, Holgate ST, Roche WR (1999). Growth factors secreted by bronchial epithelial cells control myofibroblast proliferation: an in vitro co-culture model of airway remodeling in asthma. Lab Invest.

[B7] Mondy JS, Lindner V, Miyashiro JK, Berk BC, Dean RH, Geary RL (1997). Platelet-derived growth factor ligand and receptor expression in response to altered blood flow in vivo. Circ Res.

[B8] Lindroos PM, Coin PG, Badgett A, Morgan DL, Bonner JC (1997). Alveolar macrophages stimulated with titanium dioxide, chrysotile asbestos, and residual oil fly ash upregulate the PDGF receptor-alpha on lung fibroblasts through an IL-1beta-dependent mechanism. Am J Respir Cell Mol Biol.

[B9] Ponten A, Li X, Thoren P, Aase K, Sjoblom T, Ostman A, Eriksson U (2003). Transgenic overexpression of platelet-derived growth factor-C in the mouse heart induces cardiac fibrosis, hypertrophy, and dilated cardiomyopathy. Am J Pathol.

[B10] Pinzani M, Milani S, Herbst H, DeFranco R, Grappone C, Gentilini A, Caligiuri A, Pellegrini G, Ngo DV, Romanelli RG, Gentilini P (1996). Expression of platelet-derived growth factor and its receptors in normal human liver and during active hepatic fibrogenesis. Am J Pathol.

[B11] Iida H, Seifert R, Alpers CE, Gronwald RG, Phillips PE, Pritzl P, Gordon K, Gown AM, Ross R, Bowen-Pope DF, . (1991). Platelet-derived growth factor (PDGF) and PDGF receptor are induced in mesangial proliferative nephritis in the rat. Proc Natl Acad Sci U S A.

[B12] Wilcox JN, Smith KM, Williams LT, Schwartz SM, Gordon D (1988). Platelet-derived growth factor mRNA detection in human atherosclerotic plaques by in situ hybridization. J Clin Invest.

[B13] Rice AB, Moomaw CR, Morgan DL, Bonner JC (1999). Specific inhibitors of platelet-derived growth factor or epidermal growth factor receptor tyrosine kinase reduce pulmonary fibrosis in rats. Am J Pathol.

[B14] Yagi M, Kato S, Kobayashi Y, Kobayashi N, Iinuma N, Nakamura K, Kubo K, Ohyama SI, Murooka H, Shimizu T, Nishitoba T, Osawa T, Nagano N (1998). Beneficial effects of a novel inhibitor of platelet-derived growth factor receptor autophosphorylation in the rat with mesangial proliferative glomerulonephritis. Gen Pharmacol.

[B15] Heinrich MC, McArthur GA, Demetri GD, Joensuu H, Bono P, Herrmann R, Hirte H, Cresta S, Koslin DB, Corless CL, Dirnhofer S, van Oosterom AT, Nikolova Z, Dimitrijevic S, Fletcher JA (2006). Clinical and molecular studies of the effect of imatinib on advanced aggressive fibromatosis (desmoid tumor). J Clin Oncol.

[B16] Adams EF, Todo T, Schrell UM, Thierauf P, White MC, Fahlbusch R (1991). Autocrine control of human meningioma proliferation: secretion of platelet-derived growth-factor-like molecules. Int J Cancer.

[B17] Hermanson M, Funa K, Hartman M, Claesson-Welsh L, Heldin CH, Westermark B, Nister M (1992). Platelet-derived growth factor and its receptors in human glioma tissue: expression of messenger RNA and protein suggests the presence of autocrine and paracrine loops. Cancer Res.

[B18] Nister M, Libermann TA, Betsholtz C, Pettersson M, Claesson-Welsh L, Heldin CH, Schlessinger J, Westermark B (1988). Expression of messenger RNAs for platelet-derived growth factor and transforming growth factor-alpha and their receptors in human malignant glioma cell lines. Cancer Res.

[B19] Li X, Ponten A, Aase K, Karlsson L, Abramsson A, Uutela M, Backstrom G, Hellstrom M, Bostrom H, Li H, Soriano P, Betsholtz C, Heldin CH, Alitalo K, Ostman A, Eriksson U (2000). PDGF-C is a new protease-activated ligand for the PDGF alpha-receptor. Nat Cell Biol.

[B20] Bergsten E, Uutela M, Li X, Pietras K, Ostman A, Heldin CH, Alitalo K, Eriksson U (2001). PDGF-D is a specific, protease-activated ligand for the PDGF beta-receptor. Nat Cell Biol.

[B21] Zhuo Y, Zhang J, Laboy M, Lasky JA (2004). Modulation of PDGF-C and PDGF-D expression during bleomycin-induced lung fibrosis. Am J Physiol Lung Cell Mol Physiol.

[B22] Heldin CH (1997). Simultaneous induction of stimulatory and inhibitory signals by PDGF. FEBS Lett.

[B23] Hammacher A, Mellstrom K, Heldin CH, Westermark B (1989). Isoform-specific induction of actin reorganization by platelet-derived growth factor suggests that the functionally active receptor is a dimer. EMBO J.

[B24] Kanakaraj P, Raj S, Khan SA, Bishayee S (1991). Ligand-induced interaction between alpha- and beta-type platelet-derived growth factor (PDGF) receptors: role of receptor heterodimers in kinase activation. Biochemistry.

[B25] Seifert RA, Hart CE, Phillips PE, Forstrom JW, Ross R, Murray MJ, Bowen-Pope DF (1989). Two different subunits associate to create isoform-specific platelet-derived growth factor receptors. J Biol Chem.

[B26] Schlessinger J (1993). How receptor tyrosine kinases activate Ras. Trends Biochem Sci.

[B27] Franke TF, Yang SI, Chan TO, Datta K, Kazlauskas A, Morrison DK, Kaplan DR, Tsichlis PN (1995). The protein kinase encoded by the Akt proto-oncogene is a target of the PDGF-activated phosphatidylinositol 3-kinase. Cell.

[B28] Powell DW, Mifflin RC, Valentich JD, Crowe SE, Saada JI, West AB (1999). Myofibroblasts. I. Paracrine cells important in health and disease. Am J Physiol.

[B29] Siegbahn A, Hammacher A, Westermark B, Heldin CH (1990). Differential effects of the various isoforms of platelet-derived growth factor on chemotaxis of fibroblasts, monocytes, and granulocytes. J Clin Invest.

[B30] Shih AH, Holland EC (2006). Platelet-derived growth factor (PDGF) and glial tumorigenesis. Cancer Lett.

[B31] Lokker NA, Sullivan CM, Hollenbach SJ, Israel MA, Giese NA (2002). Platelet-derived growth factor (PDGF) autocrine signaling regulates survival and mitogenic pathways in glioblastoma cells: evidence that the novel PDGF-C and PDGF-D ligands may play a role in the development of brain tumors. Cancer Res.

[B32] Todo T, Adams EF, Fahlbusch R (1993). Inhibitory effect of trapidil on human meningioma cell proliferation via interruption of autocrine growth stimulation. J Neurosurg.

[B33] Guha A, Dashner K, Black PM, Wagner JA, Stiles CD (1995). Expression of PDGF and PDGF receptors in human astrocytoma operation specimens supports the existence of an autocrine loop. Int J Cancer.

[B34] Abdelkader M, Riad M, Williams A (2001). Aggressive fibromatosis of the head and neck (desmoid tumours). J Laryngol Otol.

[B35] Heinrich MC, Corless CL, Duensing A, McGreevey L, Chen CJ, Joseph N, Singer S, Griffith DJ, Haley A, Town A, Demetri GD, Fletcher CD, Fletcher JA (2003). PDGFRA activating mutations in gastrointestinal stromal tumors. Science.

[B36] Basciani S, Brama M, Mariani S, De Luca G, Arizzi M, Vesci L, Pisano C, Dolci S, Spera G, Gnessi L (2005). Imatinib mesylate inhibits Leydig cell tumor growth: evidence for in vitro and in vivo activity. Cancer Res.

[B37] Kantarjian HM, Cortes JE, O'Brien S, Luthra R, Giles F, Verstovsek S, Faderl S, Thomas D, Garcia-Manero G, Rios MB, Shan J, Jones D, Talpaz M (2004). Long-term survival benefit and improved complete cytogenetic and molecular response rates with imatinib mesylate in Philadelphia chromosome-positive chronic-phase chronic myeloid leukemia after failure of interferon-alpha. Blood.

[B38] Melichar B, Voboril Z, Nozicka J, Ryska A, Urminska H, Vanecek T, Michal M (2005). Pathological complete response in advanced gastrointestinal stromal tumor after imatinib therapy. Intern Med.

[B39] Risau W, Drexler H, Mironov V, Smits A, Siegbahn A, Funa K, Heldin CH (1992). Platelet-derived growth factor is angiogenic in vivo. Growth Factors.

[B40] Lindahl P, Johansson BR, Leveen P, Betsholtz C (1997). Pericyte loss and microaneurysm formation in PDGF-B-deficient mice. Science.

[B41] Abramsson A, Lindblom P, Betsholtz C (2003). Endothelial and nonendothelial sources of PDGF-B regulate pericyte recruitment and influence vascular pattern formation in tumors. J Clin Invest.

[B42] Enge M, Bjarnegard M, Gerhardt H, Gustafsson E, Kalen M, Asker N, Hammes HP, Shani M, Fassler R, Betsholtz C (2002). Endothelium-specific platelet-derived growth factor-B ablation mimics diabetic retinopathy. EMBO J.

[B43] Carmeliet P, Jain RK (2000). Angiogenesis in cancer and other diseases. Nature.

[B44] Bergers G, Song S, Meyer-Morse N, Bergsland E, Hanahan D (2003). Benefits of targeting both pericytes and endothelial cells in the tumor vasculature with kinase inhibitors. J Clin Invest.

[B45] Abdollahi A, Lipson KE, Han X, Krempien R, Trinh T, Weber KJ, Hahnfeldt P, Hlatky L, Debus J, Howlett AR, Huber PE (2003). SU5416 and SU6668 attenuate the angiogenic effects of radiation-induced tumor cell growth factor production and amplify the direct anti-endothelial action of radiation in vitro. Cancer Res.

[B46] Erber R, Thurnher A, Katsen AD, Groth G, Kerger H, Hammes HP, Menger MD, Ullrich A, Vajkoczy P (2004). Combined inhibition of VEGF and PDGF signaling enforces tumor vessel regression by interfering with pericyte-mediated endothelial cell survival mechanisms. FASEB J.

[B47] Motzer RJ, Rini BI, Bukowski RM, Curti BD, George DJ, Hudes GR, Redman BG, Margolin KA, Merchan JR, Wilding G, Ginsberg MS, Bacik J, Kim ST, Baum CM, Michaelson MD (2006). Sunitinib in patients with metastatic renal cell carcinoma. JAMA.

[B48] Strumberg D (2005). Preclinical and clinical development of the oral multikinase inhibitor sorafenib in cancer treatment. Drugs Today (Barc ).

[B49] Budach W, Taghian A, Freeman J, Gioioso D, Suit HD (1993). Impact of stromal sensitivity on radiation response of tumors. J Natl Cancer Inst.

[B50] Guerry-Force ML, Perkett EA, Brigham KL, Meyrick B (1988). Early structural changes in sheep lung following thoracic irradiation. Radiat Res.

[B51] Li M, Ping G, Plathow C, Trinh T, Lipson KE, Hauser K, Krempien R, Debus J, Abdollahi A, Huber PE (2006). Small molecule receptor tyrosine kinase inhibitor of platelet-derived growth factor signaling (SU9518) modifies radiation response in fibroblasts and endothelial cells. BMC Cancer.

[B52] Kerbel R, Folkman J (2002). Clinical translation of angiogenesis inhibitors. Nat Rev Cancer.

[B53] Geng L, Donnelly E, McMahon G, Lin PC, Sierra-Rivera E, Oshinka H, Hallahan DE (2001). Inhibition of vascular endothelial growth factor receptor signaling leads to reversal of tumor resistance to radiotherapy. Cancer Res.

[B54] Holdhoff M, Kreuzer KA, Appelt C, Scholz R, Na IK, Hildebrandt B, Riess H, Jordan A, Schmidt CA, Van Etten RA, Dorken B, le Coutre P (2005). Imatinib mesylate radiosensitizes human glioblastoma cells through inhibition of platelet-derived growth factor receptor. Blood Cells Mol Dis.

[B55] Plathow C, Li M, Gong P, Zieher H, Kiessling F, Peschke P, Kauczor HU, Abdollahi A, Huber PE (2004). Computed tomography monitoring of radiation-induced lung fibrosis in mice. Invest Radiol.

[B56] Abratt RP, Morgan GW, Silvestri G, Willcox P (2004). Pulmonary complications of radiation therapy. Clin Chest Med.

[B57] Trott KR, Herrmann T, Kasper M (2004). Target cells in radiation pneumopathy. International Journal of Radiation Oncology*Biology*Physics.

[B58] Chen ES, Greenlee BM, Wills-Karp M, Moller DR (2001). Attenuation of lung inflammation and fibrosis in interferon-gamma-deficient mice after intratracheal bleomycin. Am J Respir Cell Mol Biol.

[B59] Sunyach MP, Falchero L, Pommier P, Perol M, Arpin D, Vincent M, Boutry D, Rebatu P, Ginestet C, Martel-Lafay I, Perol D, Carrie C (2000). Prospective evaluation of early lung toxicity following three-dimensional conformal radiation therapy in non-small-cell lung cancer: preliminary results. Int J Radiat Oncol Biol Phys.

[B60] Rosenzweig KE, Mychalczak B, Fuks Z, Hanley J, Burman C, Ling CC, Armstrong J, Ginsberg R, Kris MG, Raben A, Leibel S (2000). Final report of the 70.2-Gy and 75.6-Gy dose levels of a phase I dose escalation study using three-dimensional conformal radiotherapy in the treatment of inoperable non-small cell lung cancer. Cancer J.

[B61] Kong FM, Hayman JA, Griffith KA, Kalemkerian GP, Arenberg D, Lyons S, Turrisi A, Lichter A, Fraass B, Eisbruch A, Lawrence TS, Ten Haken RK (2006). Final toxicity results of a radiation-dose escalation study in patients with non-small-cell lung cancer (NSCLC): Predictors for radiation pneumonitis and fibrosis. Int J Radiat Oncol Biol Phys.

[B62] McBride WH (1995). Cytokine cascades in late normal tissue radiation responses. Int J Radiat Oncol Biol Phys.

[B63] Kamp DW (2003). Idiopathic pulmonary fibrosis: the inflammation hypothesis revisited. Chest.

[B64] Rubin P, Johnston CJ, Williams JP, McDonald S, Finkelstein JN (1995). A perpetual cascade of cytokines postirradiation leads to pulmonary fibrosis. Int J Radiat Oncol Biol Phys.

[B65] Broekelmann TJ, Limper AH, Colby TV, McDonald JA (1991). Transforming growth factor beta 1 is present at sites of extracellular matrix gene expression in human pulmonary fibrosis. Proc Natl Acad Sci U S A.

[B66] Johnston CJ, Piedboeuf B, Rubin P, Williams JP, Baggs R, Finkelstein JN (1996). Early and persistent alterations in the expression of interleukin-1 alpha, interleukin-1 beta and tumor necrosis factor alpha mRNA levels in fibrosis-resistant and sensitive mice after thoracic irradiation. Radiat Res.

[B67] Lindroos PM, Coin PG, Osornio-Vargas AR, Bonner JC (1995). Interleukin 1 beta (IL-1 beta) and the IL-1 beta-alpha 2-macroglobulin complex upregulate the platelet-derived growth factor alpha-receptor on rat pulmonary fibroblasts. Am J Respir Cell Mol Biol.

[B68] Heinzelmann F, Jendrossek V, Lauber K, Nowak K, Eldh T, Boras R, Handrick R, Henkel M, Martin C, Uhlig S, Kohler D, Eltzschig HK, Wehrmann M, Budach W, Belka C (2006). Irradiation-induced pneumonitis mediated by the CD95/CD95-ligand system. J Natl Cancer Inst.

[B69] Hill RP, Rodemann HP, Hendry JH, Roberts SA, Anscher MS (2001). Normal tissue radiobiology: from the laboratory to the clinic. Int J Radiat Oncol Biol Phys.

[B70] Tada H, Ogushi F, Tani K, Nishioka Y, Miyata J, Sato K, Asano T, Sone S (2003). Increased Binding and Chemotactic Capacities of PDGF-BB on Fibroblasts in Radiation Pneumonitis. Radiation Research.

[B71] Bonner JC, Goodell AL, Coin PG, Brody AR (1993). Chrysotile asbestos upregulates gene expression and production of alpha-receptors for platelet-derived growth factor (PDGF-AA) on rat lung fibroblasts. J Clin Invest.

[B72] Gurujeyalakshmi G, Hollinger MA, Giri SN (1996). Inhibitory effect of interferon gamma, interleukin-1, interleukin-6 and platelet-derived growth factor-A mRNA expression in bleomycin-mouse model of lung fibrosis.. Res Commun Pharmacol Toxicol.

[B73] Liu JY, Morris GF, Lei WH, Hart CE, Lasky JA, Brody AR (1997). Rapid activation of PDGF-A and -B expression at sites of lung injury in asbestos-exposed rats. Am J Respir Cell Mol Biol.

[B74] Hoyle GW, Li J, Finkelstein JB, Eisenberg T, Liu JY, Lasky JA, Athas G, Morris GF, Brody AR (1999). Emphysematous lesions, inflammation, and fibrosis in the lungs of transgenic mice overexpressing platelet-derived growth factor. Am J Pathol.

[B75] Yoshida M, Sakuma J, Hayashi S, Abe K, Saito I, Harada S, Sakatani M, Yamamoto S, Matsumoto N, Kaneda Y, . (1995). A histologically distinctive interstitial pneumonia induced by overexpression of the interleukin 6, transforming growth factor beta 1, or platelet-derived growth factor B gene. Proc Natl Acad Sci U S A.

[B76] Duan DS, Pazin MJ, Fretto LJ, Williams LT (1991). A functional soluble extracellular region of the platelet-derived growth factor (PDGF) beta-receptor antagonizes PDGF-stimulated responses. J Biol Chem.

[B77] Ferns GA, Raines EW, Sprugel KH, Motani AS, Reidy MA, Ross R (1991). Inhibition of neointimal smooth muscle accumulation after angioplasty by an antibody to PDGF. Science.

[B78] Abdollahi A, Li M, Ping G, Plathow C, Domhan S, Kiessling F, Lee LB, McMahon G, Grone HJ, Lipson KE, Huber PE (2005). Inhibition of platelet-derived growth factor signaling attenuates pulmonary fibrosis. J Exp Med.

[B79] Heldin CH, Westermark B (1999). Mechanism of action and in vivo role of platelet-derived growth factor. Physiol Rev.

[B80] Blatti SP, Foster DN, Ranganathan G, Moses HL, Getz MJ (1988). Induction of fibronectin gene transcription and mRNA is a primary response to growth-factor stimulation of AKR-2B cells. Proc Natl Acad Sci U S A.

[B81] Heldin P, Laurent TC, Heldin CH (1989). Effect of growth factors on hyaluronan synthesis in cultured human fibroblasts. Biochem J.

[B82] Canalis E (1981). Effect of platelet-derived growth factor on DNA and protein synthesis in cultured rat calvaria. Metabolism.

[B83] Schonherr E, Jarvelainen HT, Sandell LJ, Wight TN (1991). Effects of platelet-derived growth factor and transforming growth factor-beta 1 on the synthesis of a large versican-like chondroitin sulfate proteoglycan by arterial smooth muscle cells. J Biol Chem.

[B84] Zerwes HG, Risau W (1987). Polarized secretion of a platelet-derived growth factor-like chemotactic factor by endothelial cells in vitro. J Cell Biol.

[B85] Antoniades HN, Bravo MA, Avila RE, Galanopoulos T, Neville-Golden J, Maxwell M, Selman M (1990). Platelet-derived growth factor in idiopathic pulmonary fibrosis. J Clin Invest.

[B86] Dik WA, Versnel MA, Naber BA, Janssen DJ, van Kaam AH, Zimmermann LJ (2003). Dexamethasone treatment does not inhibit fibroproliferation in chronic lung disease of prematurity. Eur Respir J.

[B87] Savikko J, Taskinen E, Von Willebrand E (2003). Chronic allograft nephropathy is prevented by inhibition of platelet-derived growth factor receptor: tyrosine kinase inhibitors as a potential therapy. Transplantation.

[B88] Nicod LP (1999). Pirfenidone in idiopathic pulmonary fibrosis. Lancet.

[B89] Ziesche R, Hofbauer E, Wittmann K, Petkov V, Block LH (1999). A preliminary study of long-term treatment with interferon gamma-1b and low-dose prednisolone in patients with idiopathic pulmonary fibrosis. N Engl J Med.

[B90] Gurujeyalakshmi G, Hollinger MA, Giri SN (1999). Pirfenidone inhibits PDGF isoforms in bleomycin hamster model of lung fibrosis at the translational level. Am J Physiol.

